# A living systematic review protocol for COVID-19 clinical trial registrations

**DOI:** 10.12688/wellcomeopenres.15821.1

**Published:** 2020-04-02

**Authors:** Brittany J. Maguire, Philippe J. Guérin

**Affiliations:** 1 Infectious Diseases Data Observatory (IDDO), Oxford, UK; 2 Centre for Tropical Medicine and Global Health, Nuffield Department of Medicine, University of Oxford, Oxford, UK

**Keywords:** Living systematic review, COVID-19, coronavirus, clinical trials, emerging infections

## Abstract

Since the coronavirus disease 2019 (COVID-19) outbreak was identified in December 2019 in Wuhan, China, a strong response from the research community has been observed with the proliferation of independent clinical trials assessing diagnostic methods, therapeutic and prophylactic strategies. While there is no intervention for the prevention or treatment of COVID-19 with proven clinical efficacy to date, tools to distil the current research landscape by intervention, level of evidence and those studies likely powered to address future research questions is essential.

This living systematic review aims to provide an open, accessible and frequently updated resource summarising the characteristics of COVID-19 clinical trial registrations. Weekly search updates of the WHO International Clinical Trials Registry Platform (ICTRP) and source registries will be conducted. Data extraction by two independent reviewers of trial characteristic variables including categorisation of trial design, geographic location, intervention type and targets, level of evidence and intervention adaptability to low resource settings will be completed. Descriptive and thematic synthesis will be conducted.

A searchable and interactive visualisation of the results database will be created, and made openly available online. Weekly results from the continued search updates will be published and made available on the Infectious Diseases Data Observatory (IDDO) website ( COVID-19 website).

This living systematic review will provide a useful resource of COVID-19 clinical trial registrations for researchers in a rapidly evolving context. In the future, this sustained review will allow prioritisation of research targets for individual patient data meta-analysis.

## Introduction

The urgency of the international response to the COVID-19 pandemic has challenged the research community’s coordination and collaboration, resulting in hundreds of independent efforts to test and trial interventions. As new clinical trials are being registered every week, if not on a daily basis, a frequently updated resource is required to aid in distilling the many diagnostic methods, therapeutic and prophylactic strategies being assessed and to provide clarity around those trials that are well designed and powered to identify small benefits for promptly addressing critical research questions.

All countries share the common constraint of a finite budget and resources in combating pandemics, as is currently being witnessed with COVID-19 ^1^. Yet not all have the same basic level of health infrastructure to deal with day-to-day health emergencies, let alone global health threats of this magnitude ^2^. To date, a comparatively small number of confirmed COVID-19 cases have been reported in regions with low resource settings (including Africa, Latin America and South Asia). However, as cases are expected to rise over the coming weeks those countries with resource constrained health care systems will be disproportionately affected ^3^. If proven effective, readily implementable and scalable interventions are demanded. A means for researchers to rapidly categorise and identify priority trials assessing interventions that are affordable, readily available and adaptable particularly to low resource settings could assist this effort.

Furthermore, it is expected that resource limited countries will conduct research studies which will respond to their contexts and duplication of effort should be minimised as much as possible. While, to date, there is no available vaccine or drug with proven clinical efficacy, a method for describing ongoing trials as well as extracting pertinent information for each of them is essential within the current research landscape.

To address this need, a living systematic review ^4^ will be established to continually update and incorporate new clinical trial registrations and relevant data as they become available. The primary objective of this living systematic review is to provide an open, accessible and frequently updated resource summarising the characteristics of COVID-19 clinical trial registrations.

## Methods

The living systematic review protocol outlined herein was prospectively designed. Due to the time sensitivity of this project being undertaken during a pandemic, preliminary data extraction necessarily commenced before it could be formally registered with PROSPERO.

### Rationale for use of living method

In the setting of a current pandemic with new clinical trials being registered every week, if not on a daily basis, there are limitations to conducting a systematic review at a single time point. The most substantial limitation being that systematic reviews are time intensive. The average time to write and publish a systematic review is 67 weeks ^5^. A living systematic review methodology permits minimal compromise to methodological rigour while allowing improvement in the currency, relevance, and usefulness of a systematic review ^6^. Technology can be used to semi-automate arduous processes like data extraction. These tools can facilitate significantly faster extraction of relevant data, stimulating rapid and maintained synthesis of evidence for the benefit of researchers and policy makers ^5^.

### Eligibility criteria

All clinical trial registrations either planning to, or have enrolled patients of all ages diagnosed with COVID-19 will be eligible for inclusion in this review. Additional populations of interest include any trials enrolling healthy volunteers, healthcare workers or other patient populations where health related outcomes are assessed in the context of COVID-19. This review is not limited by intervention given the desire to capture all clinical trial registrations planning to or currently evaluating any COVID-19 diagnostic, prevention or treatment modality. Furthermore, the search strategy is not restricted by outcomes, animal studies will be excluded and no limitations on language or study design will be applied.

### Information sources & Search strategy

The consistent information source for this living systematic review will be the WHO International Clinical Trials Registry Platform (ICTRP) and source registries ^7^. The WHO ICTRP will be searched every week from the start of database records (to capture ongoing updates of existing records) to the present date for each search, without limitations using the following search terms ((COVID-19) OR (coronav*) OR (*CoV-2) OR (nCoV*)). While many living systematic reviews are updated on a monthly basis ^6^, a weekly update has been selected due to the rapidly evolving context of COVID-19 and due to scheduled WHO ICTRP updates occurring weekly ^7^. For all included trials, the source clinical trial registry database will be searched and the related trial record identified in order to supplement data extraction. A number of clinical trial registry databases ^a^ are only imported to WHO ICTRP on a monthly basis. This living systematic review protocol will be iteratively revised and manual searches of some databases on a weekly basis may supplement WHO ICTRP weekly searches. Grey literature searching will also be conducted for technical or research reports of planned, active or completed clinical trials from industry, international and government agencies, and scientific research groups. Furthermore, as part of the Infectious Diseases Data Observatory (IDDO) and International Severe Acute Respiratory and emerging Infection Consortium (ISARIC) ^8^ collaborative networks, additional trials of relevance may be identified and included through communications with our global research partners and through participation in a coalition to accelerate research on the prevention and treatment of COVID-19 in low resource settings ^3^.

### Study records, data items and outcomes

Data for all eligible trial records will be extracted in a standardised, pre-piloted REDCap ^9, 10^ database. After each weekly search update, the search returns from WHO ICTRP will be exported into Trifacta ^11^ software, where a pre-piloted script will be run to wrangle the relevant variables and available data for extraction in alignment with the REDCap data dictionary before import to the REDCap database. This process will automate data entry of all available data fields from the WHO ICTRP export into the desired controlled terminology fields in the REDCap database. Additionally, it will aid in the de-duplication process and identify any updates to data fields in the records of existing clinical trial records.

Any newly imported clinical trial registration records following each weekly update will be screened by two blinded reviewers to assess eligibility for inclusion and a third reviewer used if discrepancies arise. Ineligible trial records will be deleted from the database, however any relevant trials that are cancelled after registration will be retained and marked accordingly. Following these processes, data entry of additional manual variables in REDCap will be completed by one reviewer and each variable cross-checked for quality control by a second reviewer. At least one of the two reviewers per record will be a medical doctor. Any discrepancies will be resolved by discussion and a third reviewer where necessary.

Among other study characteristic variables including trial design and geographical location, this review plans to classify and categorise interventions assessed, trial eligibility and patient characteristics, and planned outcome measures. Furthermore, a prioritisation system designed by a group of clinical research consultants will be used to clarify the research landscape by intervention, level of evidence and those studies likely powered to address future research questions. Moreover, this prioritisation system will include a categorisation of trials assessing affordable and readily available interventions for deployment in low resources settings that are feasibly adaptable to such health care systems.

### Data synthesis

Given this review will identify clinical trial records prior to published results and outcome data availability, in addition to the anticipated heterogeneity of registration data, only descriptive and thematic analysis of study characteristics will be conducted and R statistical computing software will be used for synthesis where possible.

The REDCap database for this project will be locked on 3 ^rd^ April 2020, and data from all eligible records will be included in the baseline review and initial synthesis of clinical trial registration characteristics.

A searchable and interactive visualisation of IDDO’s COVID-19 clinical trial database will be created, made openly available online and subsequently updated following the planned weekly search updates. Following each search update, data will be exported directly from the REDCap database and variables will be processed in accordance with a standard operating procedure, mapping coded categorical and binary terms to their associated descriptive terms for website display.

### Risk of bias, Meta-biases & Confidence in cumulative evidence

This living systematic review is intended to provide a frequently updated and open resource for characterising COVID-19 clinical trial registrations. Specific clinical questions will not be addressed by this systematic review, and it will be conducted only so far as data extraction, descriptive analysis of trial characteristics and visualisation of clinical trial registry records. Accordingly, there is no plan to assess meta-biases or the strength of the body of evidence represented by included records. The risk of bias of individual studies will however, be assessed at the study level using the Oxford Centre for Evidence-Based Medicine 2011 Levels of Evidence ^12^.

## Dissemination of information

Results of the baseline review will be published in an open source, peer-reviewed journal and weekly updates for the maintenance of this living systematic review will continue for the foreseeable future during 2020 on the Infectious Diseases Data Observatory (IDDO) ^13^. The protocol for sustained updates will be revised monthly and adapted as necessary in accordance with the changing context of the COVID-19 pandemic. At a later stage, this living review will allow prioritisation of research targets for individual patient data meta-analysis and will support the COVID-19 Clinical Research Coalition efforts ^3^.

As per the objectives of this systematic review and IDDO’s commitment to the FAIR principles ^14^, on completion of the baseline review, all materials related to this project will be made openly accessible on the IDDO COVID site ^13^, including the study protocol, export of the REDCap database and associated variable and data dictionaries.

## Study status

At the time of protocol submission the preliminary searches, piloting of the study selection process and formal searches for the baseline review results have been completed. The REDCap database has been established and the variable dictionary and data extraction is currently being piloted and tested.

## Data availability

### Underlying data

No data are associated with this article.

### Extended data

Extended data related to this project including data dictionary, variable dictionary and custom scripts will be made available at the below DOI when finalised.

### Reporting guidelines

Open Science Framework: PRISMA-P checklist for ‘A living systematic review protocol for COVID-19 clinical trial registrations’ https://doi.org/10.17605/OSF.IO/9MDK8 ^15^

## Footnotes

^a^Brazilian Clinical Trials Registry (ReBec), Clinical Trials Registry - India, Republic of Korea, Cuban Public Registry of Clinical Trials, German Clinical Trials Register, Iranian Registry of Clinical Trials, Japan Primary Registries Network, Pan African Clinical Trial Registry, Sri Lanka Clinical Trials Registry, Thai Clinical Trials Registry (TCTR), Peruvian Clinical Trials Registry (REPEC)
