# Supplementary material for: A living systematic review protocol for COVID-19 clinical trial registrations
Source: Wellcome Open Res. 2020 Apr 2;5:60. [Version 1] doi: 10.12688/wellcomeopenres.15821.1 (PMC7141164; doi:10.12688/wellcomeopenres.15821.1)
Supplement: Peer review history

# Peer review: Reviewer response for version 1

**DOI:** 10.21956/wellcomeopenres.17353.r38328

**Published:** 2020-04-07

**Authors:** Carol H. Sibley

**Affiliations:** 1 Department of Genome Sciences, University of Washington, Seattle, WA, USA

In the heat of the COVID-19 pandemic, many different potential treatments have been tried on small numbers of patients, and some results have been promising. However, the numbers of patients have been small, and the studies differ in many important details. Now, more formal trials with larger numbers of subjects are needed to solidly define potential treatments for patients and for possible preventive strategies, vaccines or drugs. Trials are being planned already by a number of groups; the Coalition for Epidemic Preparedness Innovations (CEPI), and the SARS-CoV-2 Diagnostic Pipeline are preparing to launch. The studies will be registered by the WHO in the global SOLIDARITY trials database. Clearly, things are moving fast. Having a single place to find the details from each study - the protocols used, patient characteristics, drugs or vaccines administered and, when finished, the outcomes would be extremely useful.

 The paper by Maguire and Guérin proposes an organized system for creation and maintenance of exactly such a system. There will be two steps. First, basic information on the plans for all trials being registered with the WHO International Clinical Trials Registry Platform (ICTRP) will be identified. The weekly systematic review of all published literature on COVID-19 and the causative virus, CoV-2 will identify new entries. The source documents will be located and a defined decision system used to identify those that describe formal trials of COVID-19 patient treatment or prevention in populations at risk of infection. 

 The living database will then have a single organized place where the range of clinical trials of drugs or vaccines being undertaken can be accessed in detail at this early stage.

 There is a strong agreement among researchers studying COVID-19 disease that data from these clinical trials will be shared openly when each trial is completed. The second step of the living reviews will then access the shared data and use rapid electronic data management tools to store details of the results in a data structure that is uniform. As trials are completed, this approach will assure that results from the various trials will be available in a common format, facilitating comparisons among trial outcomes in similar studies with a far larger number of subjects.

 The living review is well planned, uses protocols designed to access and assess published studies in a systematic, unbiased way. The result will be a very complete record of the outcomes of the numerous trials currently planned and already underway. The authors report that an initial baseline review has already identified 332 COVID-19 related studies that fulfill the established criteria for inclusion in the repository.

 In this fast moving field, the weekly review will match this rapid growth and the additional relevant studies will provide a growing foundation of solid information collected worldwide. With this tool, protocols and outcomes on treatment and prevention of COVID-19 can be rapidly compared among large number of subjects from different locations. Safe and effective drugs for treatment and vaccines or drugs for prevention can be identified. Appropriate guidelines can be established to assure that treatments and prevention protocols can be effective and safe in people of all ages and health status wherever they live. 

 This virus is already found worldwide, and the treatments must be available and effective globally, as well. This database can establish a foundation for assuring that outcome.

Is the study design appropriate for the research question?

Yes

Is the rationale for, and objectives of, the study clearly described?

Yes

Are sufficient details of the methods provided to allow replication by others?

Yes

Are the datasets clearly presented in a useable and accessible format?

Not applicable

Reviewer Expertise:

Clinical trials of Malaria drug efficacy and safety.

I confirm that I have read this submission and believe that I have an appropriate level of expertise to confirm that it is of an acceptable scientific standard.

# Peer review: Reviewer response for version 1

**DOI:** 10.21956/wellcomeopenres.17353.r38348

**Published:** 2020-04-06

**Authors:** Julia C. Cutts

**Affiliations:** 1 Burnet Institute, Melbourne, Australia

1. The study protocol by Maguire and Guerin clearly outlines the rationale and objectives for a living systematic review of COVID-19 clinical trial registrations. Briefly, the aim of the review is to provide an open resource summarizing the characteristics of COVID-19 clinical trial registrations, which will be updated on a weekly basis.
2. The choice of a living systematic review design, which involves continual updates, is appropriate and has been well-justified by the authors. In particular, a living systematic review approach is highly warranted in the context of COVID-19 clinical research because it is an incredibly fast-evolving field, with new trials being registered on a daily to weekly basis. Importantly, the study protocol adheres to the PRISMA-P guidelines.
3. The methods were well-written and contained most of the detail necessary to allow replication by others. However, I would like to highlight the following areas that warrant further clarification:
  a) Among COVID-19 those “trials” currently listed on the WHO International Clinical Trials Registry Platform (ICTRP) database (excel output) are studies classified as “observational studies” under the “Study type” variable. Will these studies be included or excluded from the systematic review? If trials listed on the ICTRP database are to be screened for inclusion against this or other criteria, then this should be specified.
  b) How will missing data be handled? Can the authors confirm whether or not they plan to contact trial investigators to request missing information where it is incomplete or ambiguous on ICTRP database?

Additional minor points:

- The final line of the first paragraph of the abstract was a little unclear:
  “While there is no intervention for the prevention or treatment of COVID-19 with proven clinical efficacy to date, tools to distil the current research landscape by intervention, level of evidence and those studies likely powered to address future research questions is essential.”
  Do the authors mean we need tools to distill the current research landscape by intervention, level of evidence and by the likelihood that the study is powered to address the research question being addressed in the study.
- Similarly, in the section ‘Study records, data items and outcomes’ the authors specify that a prioritization system will be used to clarify the research landscape by intervention, level of evidence and those studies likely powered to address future research questions. What do the authors mean by ‘future research questions’ in this context? Do the authors mean research questions in addition to those being addressed in the registered trial?

Is the study design appropriate for the research question?

Yes

Is the rationale for, and objectives of, the study clearly described?

Yes

Are sufficient details of the methods provided to allow replication by others?

Partly

Are the datasets clearly presented in a useable and accessible format?

Not applicable

Reviewer Expertise:

Systematic reviews, immuno-epidemiology, acquired and innate immunity to malaria, clinical trial protocol development.

I confirm that I have read this submission and believe that I have an appropriate level of expertise to confirm that it is of an acceptable scientific standard.
